# Forecasting COVID-19 Hospital Census: A Multivariate Time-Series Model Based on Local Infection Incidence

**DOI:** 10.2196/28195

**Published:** 2021-08-04

**Authors:** Hieu M Nguyen, Philip J Turk, Andrew D McWilliams

**Affiliations:** 1 Center for Outcomes Research and Evaluation Atrium Health Charlotte, NC United States

**Keywords:** COVID-19, forecasting, time-series model, vector error correction model, hospital census, hospital resource utilization, infection incidence

## Abstract

**Background:**

COVID-19 has been one of the most serious global health crises in world history. During the pandemic, health care systems require accurate forecasts for key resources to guide preparation for patient surges. Forecasting the COVID-19 hospital census is among the most important planning decisions to ensure adequate staffing, number of beds, intensive care units, and vital equipment.

**Objective:**

The goal of this study was to explore the potential utility of local COVID-19 infection incidence data in developing a forecasting model for the COVID-19 hospital census.

**Methods:**

The study data comprised aggregated daily COVID-19 hospital census data across 11 Atrium Health hospitals plus a virtual hospital in the greater Charlotte metropolitan area of North Carolina, as well as the total daily infection incidence across the same region during the May 15 to December 5, 2020, period. Cross-correlations between hospital census and local infection incidence lagging up to 21 days were computed. A multivariate time-series framework, called the vector error correction model (VECM), was used to simultaneously incorporate both time series and account for their possible long-run relationship. Hypothesis tests and model diagnostics were performed to test for the long-run relationship and examine model goodness of fit. The 7-days-ahead forecast performance was measured by mean absolute percentage error (MAPE), with time-series cross-validation. The forecast performance was also compared with an autoregressive integrated moving average (ARIMA) model in the same cross-validation time frame. Based on different scenarios of the pandemic, the fitted model was leveraged to produce 60-days-ahead forecasts.

**Results:**

The cross-correlations were uniformly high, falling between 0.7 and 0.8. There was sufficient evidence that the two time series have a stable long-run relationship at the .01 significance level. The model had very good fit to the data. The out-of-sample MAPE had a median of 5.9% and a 95th percentile of 13.4%. In comparison, the MAPE of the ARIMA had a median of 6.6% and a 95th percentile of 14.3%. Scenario-based 60-days-ahead forecasts exhibited concave trajectories with peaks lagging 2 to 3 weeks later than the peak infection incidence. In the worst-case scenario, the COVID-19 hospital census can reach a peak over 3 times greater than the peak observed during the second wave.

**Conclusions:**

When used in the VECM framework, the local COVID-19 infection incidence can be an effective leading indicator to predict the COVID-19 hospital census. The VECM model had a very good 7-days-ahead forecast performance and outperformed the traditional ARIMA model. Leveraging the relationship between the two time series, the model can produce realistic 60-days-ahead scenario-based projections, which can inform health care systems about the peak timing and volume of the hospital census for long-term planning purposes.

## Introduction

SARS-CoV-2 is a novel member of the coronavirus family, and infections in humans can result in the disease COVID-19. The virus is transmitted primarily through droplets from coughing and sneezing and is highly infectious. Its basic reproduction rate is estimated to be in the low to mid 2s based on different models [1], compared to 2 for severe acute respiratory syndrome (SARS) and 1.3 for the 2009 swine flu [2]. Moderate to severe disease typically manifests with acute hypoxemia, and can progress to acute respiratory distress syndrome, multiorgan dysfunction, and death. Furthermore, an estimated 25%-30% of patients admitted to hospitals require intensive care admission [2]. In December 2019, the first cases were recorded in Wuhan, China, with subsequent spread across the world. In early 2020, the World Health Organization declared COVID-19 to be a global health emergency [3]. At the end of December 2020, SARS-CoV-2 had resulted in over 82 million documented cases and nearly 2 million deaths [4].

Our work is motivated by the need of hospital leaders to have timely and accurate forecasts to guide planning for surges in hospital demands due to the pandemic. Adequate preparation can help prevent or mitigate strains on hospital resources that result when hospitals exceed their historical capacity. On the contrary, being caught off-guard under a pandemic can devastate the population and health care systems. For example, previous models in India suggested falsely that it had reached herd immunity, encouraging complacency and insufficient preparation; however, on May 4, 2021, there was still a reported rolling average of 378,000 cases a day, which overwhelmed hospitals and health workers and resulted in a national health crisis [5]. Thus, to a health care system, an essential tool is a model that provides short- and long-range forecasting of the number of COVID-19–positive patients who will be admitted. This COVID-19 hospital census plays a central role in planning decisions that frequently require considerable lead time, such as increasing staff, creating physical beds and rooms, and procuring vital equipment (eg, ventilators and personal protective equipment).

Prior research has demonstrated the utility of forecasting hospital demands (eg, hospital admissions, intensive care unit census, and hospital overall census) using univariate time-series models such as the autoregressive integrated moving average (ARIMA), the seasonal autoregressive integrated moving average (SARIMA), and exponential smoothing [6-8]. Another approach is to use ensemble-based modeling. For example, a hybrid of a SARIMA model and a nonlinear autoregression artificial neural network model has been used to forecast hospital admissions [9]. In another example, two separate models, a time-series model for hospital admission and a patient-level logistic regression model for hospital discharge, were combined to predict the hospital census [10]. While these examples demonstrate the powerful potential of univariate time-series and ensemble modeling, neither incorporate factors inherent to the behavior of the pandemic, which may serve as important leading indicators of hospital census, especially at times when infection rates become increasingly dynamic (eg, on the approach or descent of peak infection prevalence). To incorporate pandemic indicators into modeling requires recognition that such indicators are typically nonstationary. Consequently, while a stationary multivariate time-series model, called vector autoregression (VAR), has been successfully employed to forecast emergency department patient census by including other hospital resource indicators [11], it cannot be used in this situation. Rather, our problem will require nonstationary multivariate time-series models like the vector error correction model (VECM).

Recently, VECM has been used to forecast the demand for intensive care units during the COVID-19 pandemic by including hospital admission as a leading indicator [12]. Although hospital admission is a natural choice as a leading indicator, it has a short period of lead time (ie, hours to days) and thus, limited predictive power. A more powerful indicator for planning purposes would lead by days to weeks. We have previously used VECM to forecast COVID-19 hospital census using leading indicators from Google relative search volumes for COVID-19 testing–related terms combined with the number of people flagged as having possible COVID-19 when using an internet-based virtual health screening bot [13]. However, these COVID-19 indicators, which are based on symptoms, have limitations. For example, the symptoms of COVID-19 cannot be easily separated from other common conditions, such as the seasonal flu, and search patterns may change due to other external factors over time.

During the COVID-19 pandemic, many papers have been devoted to developing predictive models for the volume of new cases (ie, infection incidence) using various methods from time-series analyses [14-16] to advanced machine learning [17,18]. However, virtually no effort was focused on developing statistical models linking infection incidence to hospitalization. Because hospital admission typically follows the symptoms or exposure that may provoke a person to be tested by roughly 1 week, we hypothesize that at a local population level, infection incidence rates may have a stable relationship with and serve as a reliable leading indicator for the COVID-19 hospital census. In this paper, our main objective is to explore whether the local COVID-19 infection incidence and the COVID-19 hospital census can be successfully incorporated within a VECM to delivery satisfactory 7-days-ahead forecast performance and examine the application of this model to scenario-based long-term forecasting. From our experience, since there can be systematic changes due to the day of the week in a hospital time series, we will need to account for weekly seasonal effects and examine implications on short-term resource planning.

## Methods

### Time-Series Data

Atrium Health is a large, integrated health care system operating in North Carolina, South Carolina, and Georgia. In this paper, the COVID-19 hospital census (census) refers to the daily aggregate number of beds occupied by patients with COVID-19 at midnight across the subset of 11 Atrium Health hospitals in the greater Charlotte metropolitan area of North Carolina, plus a virtual hospital (Atrium Health Hospital at Home). The virtual hospital uses telemedicine to treat patients who require only a minimal level of care. The local COVID-19 infection incidence (incidence) is the aggregate daily count of new COVID-19–positive cases from 11 local counties belonging to the Cities Readiness Initiative (CRI) region, as designated by the North Carolina Department of Health and Human Services. The CRI region roughly approximates the market catchment area of these hospitals.

Using STL (seasonal and trend decomposition using Loess) time-series decomposition [19], we observed that the two time series had multiplicative weekly seasonality. We transformed both time series to achieve additive seasonality and linearize their relationship. The usual log transformation was applied to incidence. For operational purposes, the health system had previously decided to place an upper bound of 1000 patients with COVID-19 on the hospital time-series range, so we applied the following constrained log transformation so that the back-transformed census forecasts would satisfy the constraint:


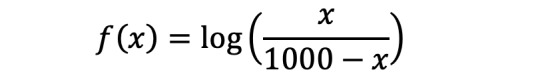


**Figure 1:**
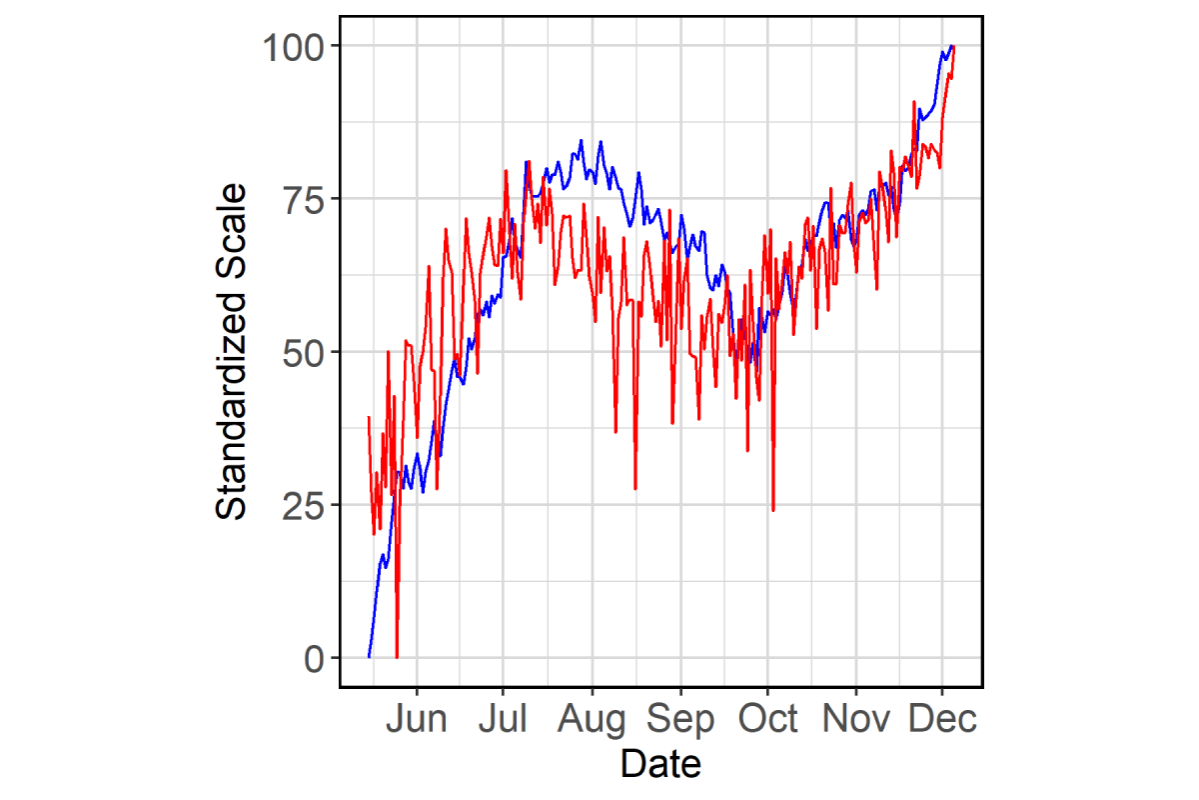
Scaled time series for COVID-19 hospital census and local COVID-19 infection incidence in the Cities Readiness Initiative region for the period from May 15 to December 5, 2020. Transformed census (blue) and incidence (red) are linearly standardized to the 0-100 scale.

The forecast model described in the following sections was developed for these transformed time series. Figure 1 shows a plot of transformed census and incidence on a standardized scale for the period from May 15 to December 5, 2020. To affirm the association between the two transformed time series, we computed the Pearson cross-correlations between census and values of incidence at lags 0, –1, …, –21.

### VECM

A VECM is a vector autoregressive model used for nonstationary multivariate time series and accounts for stable long-run relationships, that is, cointegration, between the time series. A *k* × 1 time-series vector ***y****_t_* is said to be cointegrated if there is at least one nonzero *k* × 1 vector ***β****_i_*, such that the linear combination 
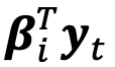
 is trend-stationary. If *r* such linearly independent vectors ***β****_i_* (*i*=1,…,*r*) exist, we say ***y****_t_* is cointegrated with cointegration rank *r* [20].

Following Pfaff [20], we first describe the VAR representation of order *p* of the VECM:


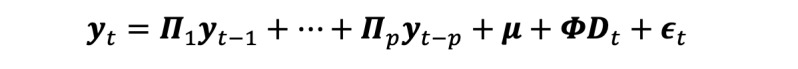


for time *t*=1,…, *T*, where ***Π****_i_* (for *i*=1,…,*p*) are *k* × *k* coefficient matrices of the lagged series at lag *i*, ***μ*** is a *k* × 1 vector of constants, ***D****_t_* is a 6 × 1 vector of weekly seasonal indicators, ***Φ*** is a *k* × 6 coefficient matrix for seasonal indicators, and ***ε****_t_* is a *k* × 1 vector of random errors.

The VECM specification can be formulated as an algebraic rearrangement of the VAR representation as:


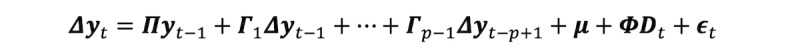


where **Δ*y****_t_* is a *k* × 1 vector of the differenced series 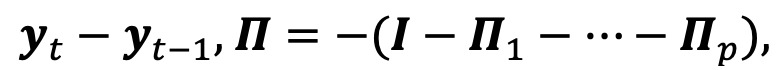 and 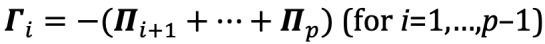.

The model has the following assumptions:

- Assumption 1: The components of ***y**_t_* are at most *I*(1), that is, an integrated of order 1
- Assumption 2: 0≤*r*=*rank*(***Π***)≤*k*
- Assumption 3: ***ε**_t_* are identically and independently distributed *N*(**0,Σ**) random vectors with covariance matrix **Σ**.

We now discuss the implications of the assumptions. For assumption 2, if *r*=*k,* then it can be shown that the VECM becomes a standard VAR model. If *r*=0, then ***Π*** is the zero matrix and there is no cointegration relationship between the series. The VECM then becomes a VAR model for differenced time series. If 0<*r*<*k*, then ***Π*** can be factored into ***Π=*** ***αβ^T^,*** where ***α*** and ***β*** are both *k* × *r* matrices. From assumption 1, the differenced series **Δ*y****_t_*, and its lags **Δ*y****_t–1,…,_* **Δ*y****_t–p+1_* are stationary. It follows that ***Πy****_t–1_*=***αβ****^T^****y****_t–1_*, as well as ***β****^T^****y****_t–1_*, also called the error correction term, is (trend-)stationary, depending on the specification of the deterministic components. The *r* linearly independent columns of ***β*** are the cointegrating vectors, and the rank *r* is equal to the cointegration rank of the system of time series.

### Estimation and Inference

The VECM was specified and fitted with the steps below.

First, to choose the order *p* of the VAR representation, we fitted a VAR model to the data and made the decision based on the Akaike information criterion (AIC) [21].

Second, we determined the number of cointegration relationships (*r*=0 or *r*=1) using the Johansen trace test [22].

Third, we needed to decide where to place the constant ***μ*** in the model. One option was to leave ***μ*** as shown previously to account for linear trend in the data. Another option was to restrict ***μ***=***αρ***. The constant would be absorbed into the cointegration relationship as an intercept, and the data would not exhibit linear trend.

We made our decision about whether to restrict ***μ*** based on a likelihood ratio test for linear trend, as described elsewhere [23,24].

Fourth, we used maximum likelihood estimation to fit the model, reported parameter estimates, the corresponding *T* tests, and the omnibus *F* tests with a significance level of .05, following Johansen [23].

Finally, we computed the 7-days-ahead forecasts and the 80% forecast intervals. Once the forecasts of the transformed census were made with the VECM, they were back-transformed to the original scale of census. We created 80% forecast intervals for the transformed census using a bootstrap procedure [25]. Then, the lower and upper bound of the forecast intervals were also back-transformed.

The model was fitted to the data between May 15 and December 5, 2020. All the data analysis was done using R statistical software, version 4.0.3 (R Core Team). The implementation of the VECM was done with the *tsDyn*, *vars*, and *urca* R packages. Since there were no packages to make bootstrapped forecast intervals for the VECM, we coded our own implementation. The data and code used in the data analysis are publicly available on GitHub [26].

### Model Diagnostics

We examined the omnibus *F* tests to look for signs of lack of fit and also performed the multivariate Portmanteau test for the existence of serial correlation in the errors. Autocorrelation function and cross-correlation function plots were also generated for visual inspection. We performed the univariate and multivariate Jarque-Bera normality test on the errors [27] and also checked whether the cointegration relationship was stable, that is, stationary, using the Augmented Dickey-Fuller (ADF) test [28] and the Kwiatkowski-Phillips-Schmidt-Shin (KPSS) test [29]. Finally, we checked the stability of the estimated VAR representation. To do so, we looked at the companion matrix of the VAR representation and checked whether the maximum eigenvalue modulus was strictly smaller than 1, which, if true, would imply the stability of the VAR representation [30]. We also generated a trace plot of the maximum eigenvalue modulus, where the model was repeatedly fitted on a daily rolling basis, to check for the consistency of this value over time.

### Forecast Performance

We used mean absolute percentage error (MAPE) to evaluate the 7-days-ahead forecasts of census:


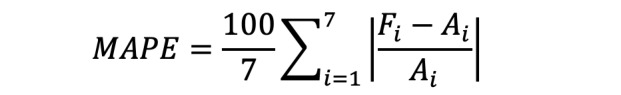


where *F_i_* is the forecast value and *A_i_* is the actual value.

In order to approximate the sampling distribution of MAPE, we performed time-series cross-validation. From June 16 to November 28, 2020, for each day, we iteratively fitted the model, made 7-days-ahead forecasts, and computed the MAPE. Eventually, we obtained 166 values of MAPE, plotted the distribution, and computed the median as well as the 95th percentile. We will consider a median MAPE below 10% to be satisfactory, based on the practical effect of a peak surge on bed capacity at our health care system.

### Scenario-Based Long-Term Forecasting

Leading up to and at the peak of infection prevalence, there can be high anxiety and uncertainty about how much more incidence and, in particular, census may increase. Furthermore, traditional univariate time-series models may give linear forecasts for census that do not accurately represent pandemic behavior. However, cointegration allows for census forecasts that leverage subtle, but critical, changes in incidence (eg, concavity). This suggests, if not necessitates, the forecasting of census under different pandemic scenarios. For resource planning, hospital leaders will want to understand the implications associated with a worst-case scenario.

**Figure 2:**
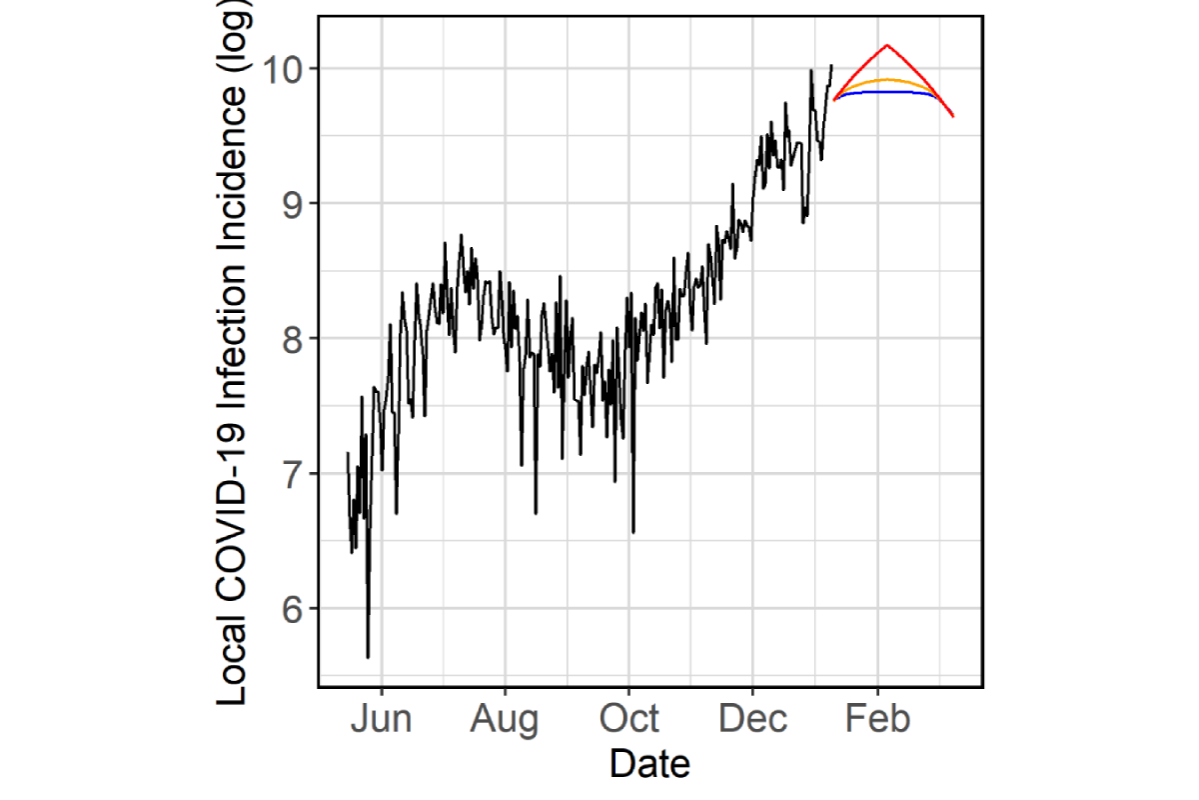
The 60-day projected local COVID-19 infection incidence in the Cities Readiness Initiative region on the log scale, as of January 9, 2021. Past values (black), worst-case scenario (red), base-case scenario (orange), best-case scenario (blue) are shown.

For our health care system, besides routine 7-days-ahead census forecasts, we also deployed our model for 60-days-ahead census forecasts, considering 3 different scenarios of what could happen with incidence (ie, best case, base case, and worst case). On January 9, 2021, we expected the winter surge to reach peak infection prevalence around February 5, 2021, based on an extension of an epidemiological model called the susceptible-infected-removed model [31]. While peak infection incidence typically leads peak infection prevalence, in the absence of definitively knowing either peak date, we took a conservative approach and linearly extrapolated incidence with a positive trend up to the expected pandemic peak. The severity of a scenario was controlled by a trend-dampening parameter [32]. After the peak, the descent path was initially symmetric to its ascent and then eventually became linear (Figure 2).

Using our model refitted on January 9, 2021, with an increased capacity of 1250 patients, we generated forecasts iteratively forward for 60 days using the past census forecasts together with projected incidence under each scenario. To account for uncertainty in future census and incidence, we also simulated 1000 conditional sample paths of the two time series under each scenario using the bootstrap procedure mentioned earlier and computed the 10th and 90th percentile at each horizon to obtain the 80% forecast intervals.

### Ethical Review

Our research protocol was submitted to the Atrium Health Institutional Review Board (IRB) prior to execution, and the study was deemed exempt from IRB oversight. In compliance with HIPAA (Health Insurance Portability and Accountability Act) regulations, individual patient information was not disclosed, and all data have been deidentified and reported as aggregates. The procedures set out in this protocol, pertaining to the conduct, evaluation, and documentation of this study, were designed to ensure that the investigators abide by Good Clinical Practice guidelines and under the guiding principles detailed in the Declaration of Helsinki.

## Results

### Estimation and Inference

**Figure 3:**
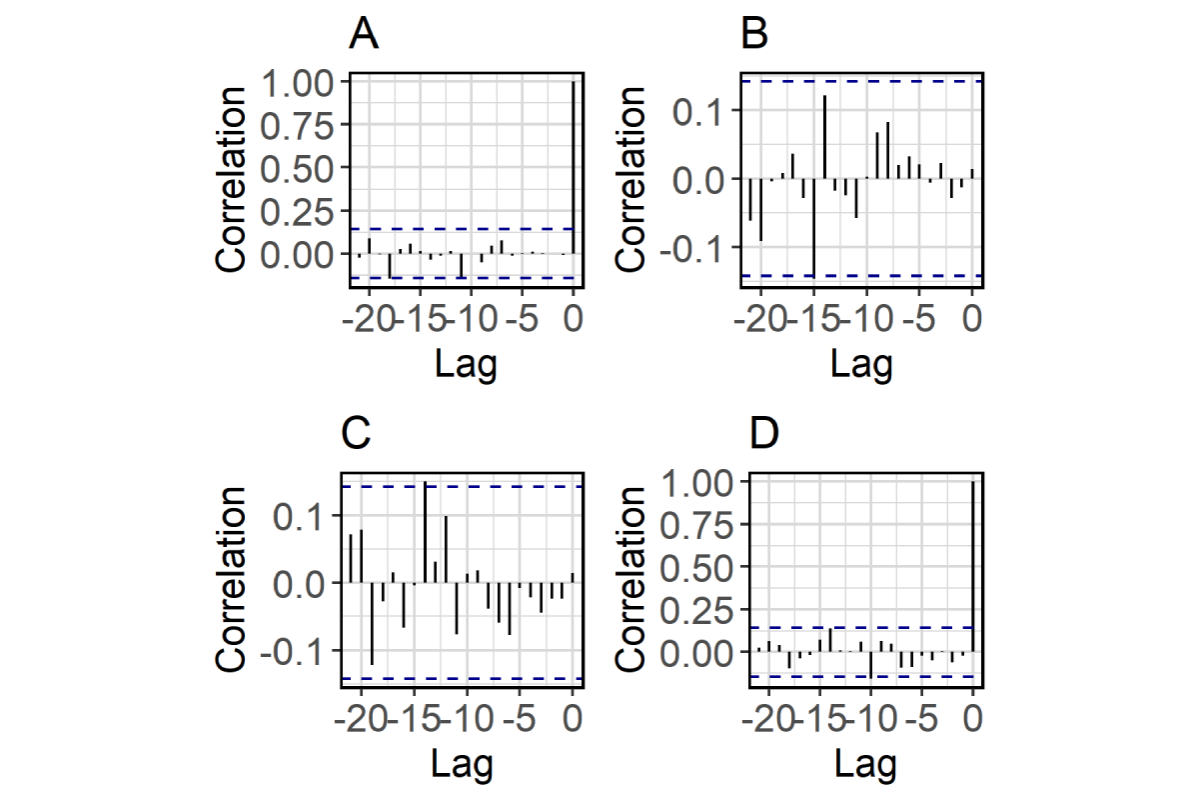
Autocorrelation functions and cross-correlation functions of the residuals: (A) census residuals, (B) lagged census residuals and incidence residuals, (C) census residuals and lagged incidence residuals, and (D) incidence residuals.

Our model was specified as a VECM with 7 lags in its VAR representation (*p*=7), 1 cointegration relationship (*r*=1), and a restricted constant parameter ***μ*** so that the series would not have linear trend. The AIC scores of VAR models with a varying number of lags from 2 to 14 were inconclusive. However, we found that 7 lags were sufficient to account for all the correlation in the data, as evidenced by the autocorrelation function and cross-correlation function plots of the residuals (Figure 3). The Johansen trace test indicated that there was 1 cointegration relationship (significant at 1%, based on tabulated critical values). Finally, the likelihood ratio test for linear trend indicated that there was no linear trend in the data (*P*=.32). Furthermore, the restricted model had a lower AIC score than the unrestricted model (the AIC scores were –1519 and –1516, respectively).

The output from the maximum likelihood estimation showed that the cointegration relationship, that is, the error correction term, had a significant negative effect on census change (*P*<.001); no significant effect was observed for incidence change (*P*=.26) (Table 1). The long-run cointegration relationship was estimated as:

*ect_t_*_–1_ =*census_t_*_–1_ – 0.8013*incidence_t_*_–1_ + 7.8266

where *ect_t_*_–1_ was the (lagged) error correction term. Table 1 also shows that past changes in census and incidence also had meaningful effects on current census change. Past census changes had significant effect at lag 2 (*P*=.002). Past incidence changes had significant effects at lag 1 (*P*=.005), lag 2 (*P*=.04), lag 4 (*P*=.02), lag 5 (*P*=.03), and lag 6 (*P*=.02).

From Table 2, there were some significant seasonal effects, that is, differences in both census and incidence changes among days of the week. Compared to Thursday, census change was higher on Monday and incidence change was lower on Sunday, with significant differences (*P*=.01 and *P*=.002, respectively).

**Table 1:** Parameter estimates and *T* tests for nonseasonal effects.

Predictor	∆*Census*_*t*_				∆*Incidence*_*t*_		
	Estimate	*T* statistics	*P* value	Estimate		*T* statistics	*P* value
*ect*_*t*–1_	–0.1265	–5.6993	<.001	–0.1216		–1.1323	.26
∆*Census*_*t*–1_	–0.0489	–0.7143	.48	0.5487		1.6555	.10
∆*Incidence*_*t*–1_	–0.0665	–2.8222	.005	–0.9808		–8.6067	<.001
∆*Census*_*t*–2_	–0.2220	–3.2277	.002	–0.0614		–0.1844	.85
∆*Incidence*_*t*–2_	–0.0532	–2.0881	.04	–0.6955		–5.6431	<.001
∆*Census*_*t*–3_	–0.0700	–0.9949	.32	0.0643		0.1890	.85
∆*Incidence*_*t*–3_	–0.0472	–1.9094	.06	–0.6428		–5.3755	<.001
∆*Census*_*t*–4_	–0.0785	–1.1224	.26	0.9769		2.8871	.004
∆*Incidence*_*t*–4_	–0.0567	–2.4165	.02	–0.5564		–4.8999	<.001
∆*Census*_*t*–5_	–0.0499	–0.7140	.48	–0.0792		–0.2341	.82
∆*Incidence*_*t*–5_	–0.0465	–2.1907	.03	–0.4589		–4.4634	<.001
∆*Census*_*t*–6_	0.0077	0.1107	.91	0.4533		1.3404	.18
∆*Incidence*_*t*–6_	–0.0373	–2.4015	.02	–0.2384		–3.1739	.002

**Table 2:** Parameter estimates and *T* tests for day-of-the-week effects, in comparison with Thursday being the reference.

Predictor	∆*Census*_*t*_				∆*Incidence*_*t*_		
	Estimate	*T* statistics	*P* value	Estimate		*T* statistics	*P* value
Friday	–0.0213	–1.1120	.27	0.0095		0.1024	.92
Saturday	0.0083	0.3980	.69	–0.1528		–1.5176	.13
Sunday	0.0030	0.1330	.89	–0.3340		–3.0744	.002
Monday	0.0585	2.6205	.01	–0.1939		–1.7950	.07
Tuesday	0.0291	1.3896	.17	–0.1284		–1.2655	.21
Wednesday	–0.0037	–0.1895	.85	0.0343		0.3672	.71

### Model Diagnostics

The omnibus *F* tests were significant for both census (*P*<.001) and incidence *P*<.001) components.

The Portmanteau test did not show sufficient evidence that the errors were autocorrelated (*P*=.19). From the residual autocorrelation function and cross-correlation function plots, the correlations were within the 95% confidence band (Figure 3). The Jarque-Bera normality tests failed to reject the normality null hypothesis for the census errors (*P*=.71) but did for incidence (*P*<.001). Specifically, the incidence residuals were moderately left-skewed. The Jarque-Bera multivariate test also rejected the multivariate normality null hypothesis (*P*<.001).

The Augmented Dickey-Fuller test for stationarity of the error correction term rejected the unit root null hypothesis at the 10% significance level but failed to reject the null hypothesis at the 5% significance level (based on tabulated critical values). The KPSS test failed to reject the stationarity null hypothesis (*P*=.10). Examination of the time plot of the predicted error correction term showed no obvious departure from stationarity.

**Figure 4:**
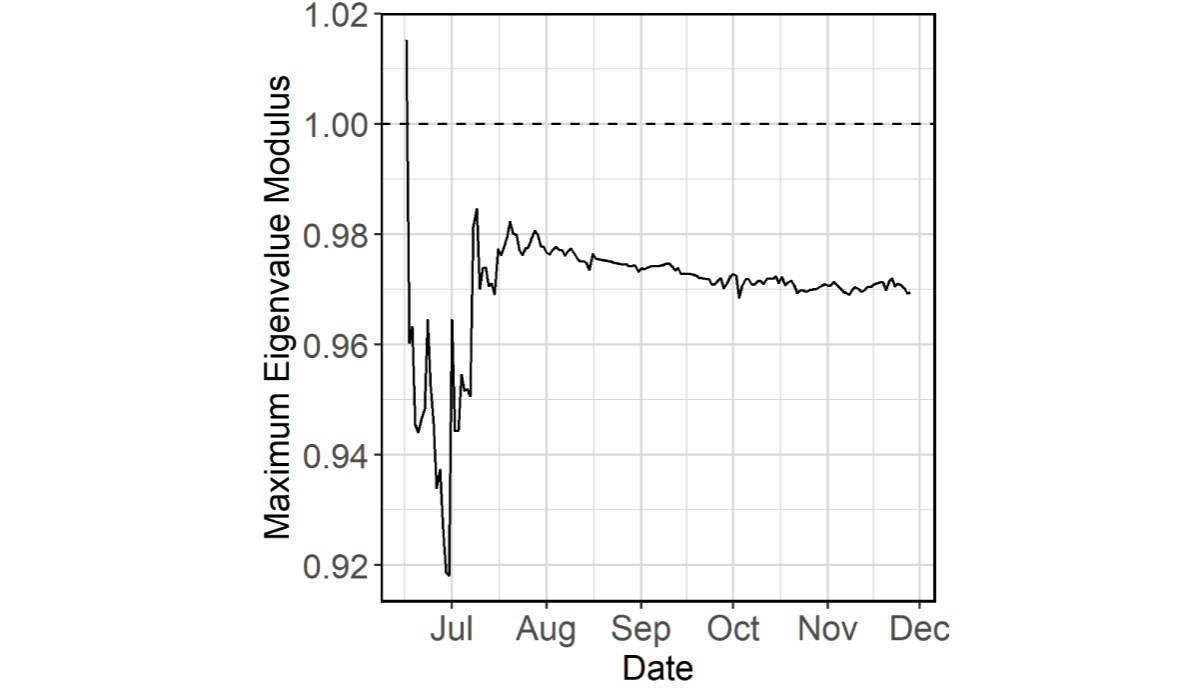
Trace plot of the maximum eigenvalue modulus for the period from June 16 to November 28, 2020.

The companion matrix of the VAR representation had a maximum eigenvalue modulus of 0.97, strictly less than 1. Although this value was close to 1, the trace plot showed that this value had been slowly declining and below 1 across time when the model was fitted repeatedly in a daily rolling basis from June 16 to November 28 (Figure 4).

### Forecast Performance

**Figure 5:**
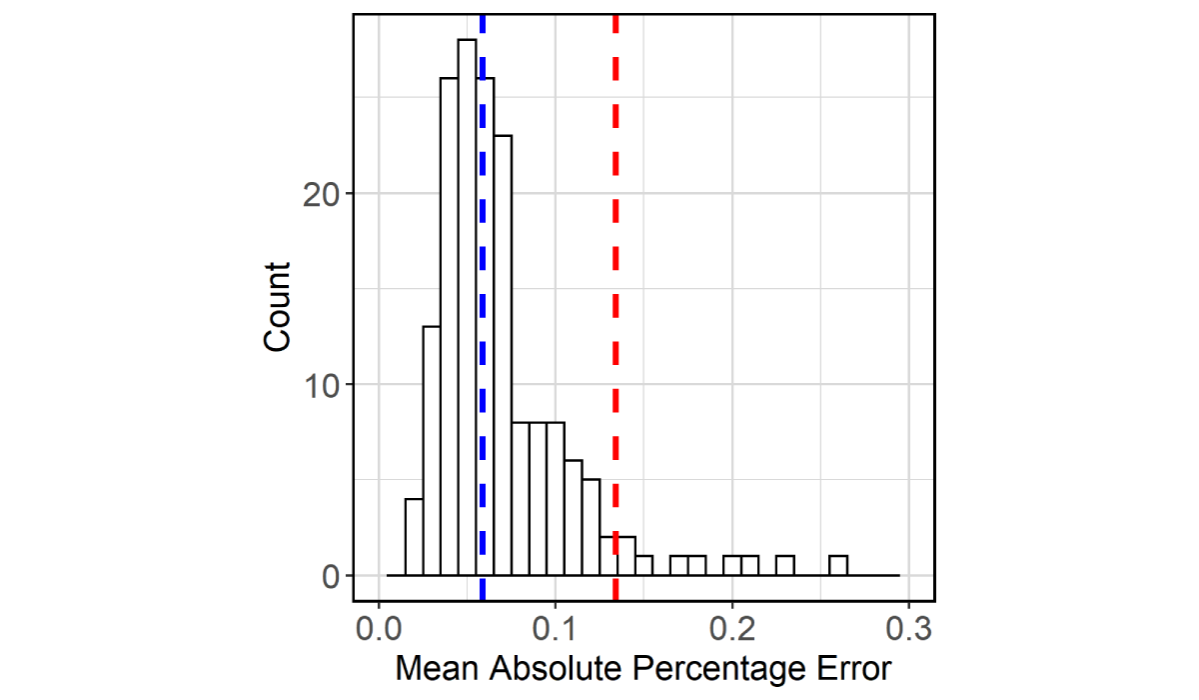
Distribution of the 7-days-ahead mean absolute percentage error from the time-series cross-validation for the period from June 16 to November 28, 2020. Median (blue) and 95th percentile (red) are shown.

**Figure 6:**
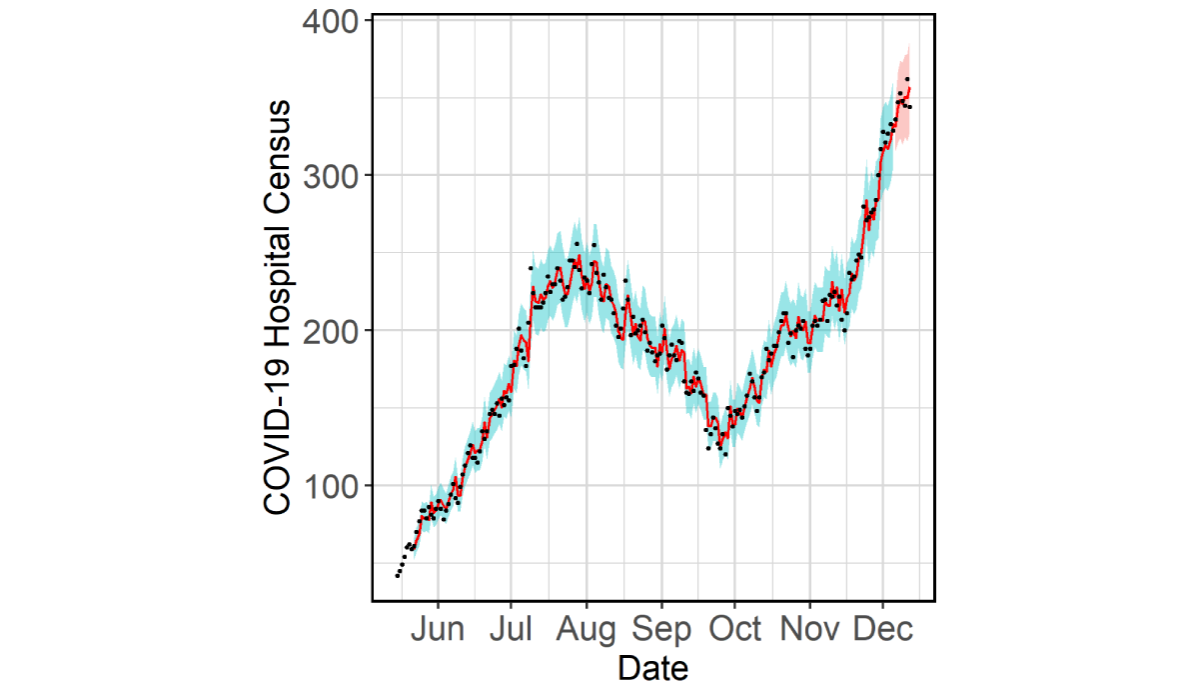
One-step-ahead in-sample and 7-days-ahead out-of-sample predictions for COVID-19 hospital census in the Cities Readiness Initiative region. True values (black), in-sample and out-of-sample predictions (red line), 95% prediction intervals (blue band), 80% forecast intervals (red band) are shown. The model is fitted on data from May 15 to December 5, 2020.

We obtained the approximate sampling distribution of the out-of-sample MAPE from the time-series cross-validation (Figure 5). The typical value (median) of MAPE was 5.9% and the 95th percentile of MAPE was 13.4%. For the sake of comparison, the corresponding values from an ARIMA model using the COVID-19 hospital census only were 6.6% and 14.3%. Additionally, after fitting the data from May 15 to December 5, we forecasted the census out to 7 days. Subsequently, the actual values were accurately forecasted with a MAPE of 1.9% and were all within the 80% bootstrapped forecast intervals (Figure 6).

### Scenario-Based Long-Term Forecasting

**Figure 7:**
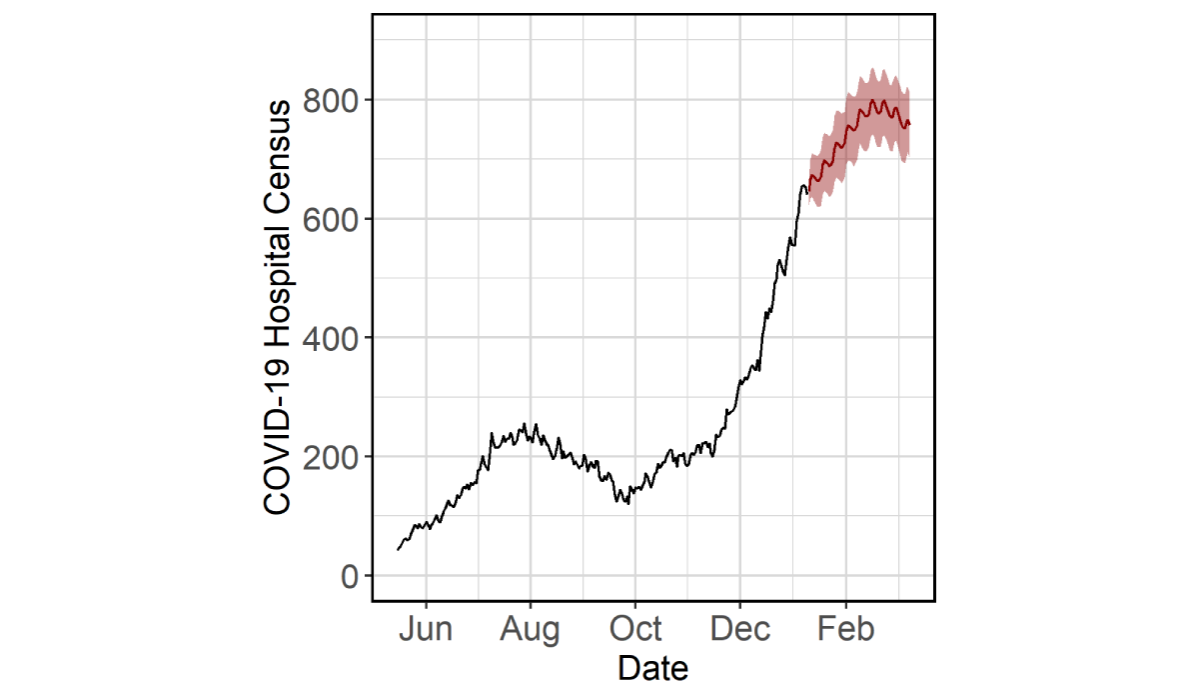
Worst-case-scenario, 60-day forecasts for COVID-19 hospital census in the Cities Readiness Initiative region, as of January 9, 2021. Past values (black), forecasts (red line), and 80% forecast intervals (red band) are shown.

In all scenarios, due to cointegration, census followed corresponding concave trajectories with peaks occurring approximately 2 to 3 weeks later than incidence depending on the scenario. In the worst-case scenario, census was projected to peak on February 16, 2021 (11 days later than incidence), with approximately 850 patients at the 80% forecast interval upper bound (Figure 7).

## Discussion

### Principal Results

Our VECM provides a very good fit to the data and outperforms models with no or other leading indicators. Significant omnibus *F* tests showed that the model fit was better than that of a reduced VECM representation with no predictors (ie, a bivariate random walk model). When we examined model diagnostics, there was no sign of any serious departure from model assumptions. From the Portmanteau test, the errors were not different from white noise (ie, the errors do not exhibit serial correlation). Although the normality assumption (for incidence) was not met, the asymptotic properties of our estimation and hypothesis tests in the VECM would not be affected [33]. To address the possible effect of this violation on the forecast intervals, we implemented a bootstrap procedure for the forecast intervals. Both the ADF test and KPSS test showed reasonable evidence that the long-run relationship was stable. With the maximum eigenvalue modulus of the VAR representation consistently below 1 across time, the model itself was quite stable. Examining the day-of-the-week effects, we observed a higher increase in census at the beginning of the week. This agrees with our observations of hospital operations and suggests higher resource allocation when starting the week, as is also reflected in the forecasts (Figure 7). In terms of forecast performance, the VECM yielded a smaller MAPE, in terms of the median and the 95th percentile, when compared to an ARIMA model using the COVID-19 hospital census only. Our VECM also performed better than another VECM that uses two internet-based leading indicators (median MAPE of 10.5%), albeit on time domains that were partially overlapping [13].

The long-run relationship plays a crucial role in the model. Our model results show how future census responds to perturbations in the long-run cointegration relationship in the direction that would preserve the stability of the relationship. For instance, if incidence increases significantly and drives the error correction term below 0, the next-day census will tend to increase so that the error correction term will move back toward 0. Compared to short-run relationships between census change and past changes in incidence and census, the long-run relationship effect is also strongly significant and is a major driver in the model.

We observed that local infection incidence led the hospital census by about 2 weeks. The cross-correlations between incidence and census were uniformly high, between 0.7 and 0.8 at different lags, but the highest correlation was at lag 14. Clinically, we know that after someone is diagnosed with SARS-CoV-2, it can take several days before they become sick enough to be hospitalized. During the summer 2020 wave of the pandemic, incidence peaked 18 days earlier, on July 10, than when census peaked, on July 28. In the model, we also saw that past incidence changes at multiple lags have statistically significant effects on census. While previous studies have focused on other types of leading indicators [12,13], our model results and our observations demonstrate that local infection incidence can be a very effective leading indicator for COVID-19 hospital census.

Applying the model to scenario-based forecasting in a health care system is an important method for long-term forecasting when approaching an infection prevalence peak and helps determine the potential for resource capacity to be exceeded under a worst-case scenario. There are several advantages to our approach. With a scenario-based and epidemiologically informed approach, the VECM produces realistic, nonlinear, long-range trajectories of census. In contrast, an ARIMA model can have an upward linear trajectory even as we approach and arrive at the infection prevalence peak because it is agnostic to incidence. Hence, the VECM fit with scenario-based incidence will provide better accuracy since it is more reflective of pandemic behavior. Additionally, when the concern is a specific scenario, our approach is particularly useful at minimizing long-range forecast uncertainty, since the bootstrapped sample paths are constrained to fluctuate around the marginalized scenario-based census projection. Without such a constraint, 60-day forecasts can typically have wide forecast intervals that are of no practical utility.

Our study has mathematically ascertained the stable long-run relationship, that is, cointegration, between the COVID-19 hospital census and the local infection incidence, and we have developed a statistical incidence-based model to forecast the COVID-19 hospital census. In comparison, prior COVID-19 hospital capacity planning models that make use of infection incidence data rely on simplified assumptions about the incidence-census relationship. For example, in the COVID-19 Hospital Impact Model for Epidemics (CHIME) at the University of Pennsylvania [34], the ratio between hospital admissions and infection incidence is a scenario parameter defined by the user and is not time varying.

### Limitations

Although our model has been thoroughly developed, it is not free of limitations. First, it is possible that we may lose the stable long-run relationship at some point in the future, either because it has run its course or due to structural changes in the time series. For instance, in the latter case, inadequate community-based testing might suddenly underestimate the actual local infection incidence, and there may be a level shift in the relationship that would have to be accounted for by a modified VECM [35,36]. In other cases, more complex structural changes may arise and be challenging to model. Second, in the future, other regions may find that the ratio between asymptomatic and symptomatic cases fluctuates considerably over time. Because case severity affects the time to hospitalization, this situation may require model revision. A potential remedy is to include both the number of asymptomatic and symptomatic cases as two leading indicators with census in a VECM in the hopes that some cointegration exists among the three variables. Third, it is relatively more difficult to fit a VECM. For univariate models such as ARIMA and exponential smoothing, well-developed R packages exist for automated model specification and estimation. With the VECM, more deliberate modeling decisions and careful checking of assumptions need to be made to fit a reliable model. Finally, the inclusion of seasonal effects in our model requires that the seasonality is deterministic. However, another health care system may find that their time-series data have stochastic seasonality or multiple deterministic seasonality. If seasonality is not important, we potentially may resolve this by simply deseasonalizing the series. Otherwise, it may be possible to account for this with more advanced parameterization of the seasonal effects.

### Conclusions

The construct presented here provides a framework in the context of a health care system for incorporating other leading indicators that may yield further increases in forecasting performance. For instance, the VECM that uses internet-based leading indicators [13] could potentially be improved by including incidence. It is also possible to incorporate other nested hospital-related time series, such as the number of intensive care units and the number of ventilators, into the VECM if there was a need to simultaneously forecast other resources. Additionally, a VECM could be a valuable candidate for a model-averaged ensemble. This can be particularly useful if the ensemble consists only of agnostic univariate time-series models.

We have shown that infection incidence can be successfully tethered with hospital census in a multivariate time-series model to achieve accurate forecasting of COVID-19 hospital census. When coupled with scenario-based forecasting, the model helped our leaders evaluate resource capacity against different possible peak resource demands. In hindsight, our analyses correctly assured our leaders of our capability to handle a worst-case scenario, alleviated uncertainty, and effectively guided long-term planning of adequate staffing, bed capacity, and equipment supplies through the pandemic.

## Abbreviations

ADF: augmented Dickey-Fuller
AIC: Akaike information criterion
ARIMA: autoregressive integrated moving average
CRI: Cities Readiness Initiative
HIPAA: Health Insurance Portability and Accountability Act
IRB: Institutional Review Board
KPSS: Kwiatkowski-Phillips-Schmidt-Shin
MAPE: mean absolute percentage error
SARIMA: seasonal autoregressive integrated moving average
SARS: severe acute respiratory syndrome
STL: seasonal and trend decomposition using Loess
VAR: vector autoregressive
VECM: vector error correction model
